# Genetic analysis of 25 Chinese pedigrees with neurofibromatosis type 1 and genotype-phenotype study from an extended cohort

**DOI:** 10.1186/s13023-025-03807-z

**Published:** 2025-05-23

**Authors:** Hongjun Fei, Xu Han, Yiyao Chen, Yan Xu, Chunxin Chang, Ming Li, Yanlin Wang, Jian Wang, Niu Li, Shuyuan Li

**Affiliations:** 1 The International Peace Maternity and Child Health Hospital, Shanghai Jiao Tong University school of Medicine, Shanghai, 200030 China; 2 Shanghai Key Laboratory of Embryo Original Diseases, Shanghai, 200030 China; 3 Faculty of Medical Laboratory Science, College of Health Science and Technology, Shanghai Jiao Tong University School of Medicine, Shanghai, 200025 China

**Keywords:** Neurofibromatosis type 1, Novel variants, Genotype–phenotype, Neurofibromas, Bone lesions

## Abstract

**Background:**

To identify the genetic variants underlying neurofibromatosis type 1 (NF1) and to investigate genotype-phenotype correlations.

**Methods:**

Thirty-three patients from 27 Chinese pedigrees with suspected NF1 phenotypes underwent genetic analysis. The impact of splicing variant on *NF1* mRNA processing was determined by cDNA direct sequencing. Additional NF1 patients with detailed clinical and molecular data were extracted from the literature for performing genotype-phenotype correlation analysis.

**Results:**

Genetic analysis identified 24 distinct *NF1* variants: nine frameshift, four nonsense, four missense, six splice site, and one exon deletion. Among them, 10 were previously unreported in the literature. A functional study showed that the canonical splicing variant (c.3497–2 A > G) resulted in an in-frame deletion of two amino acids, which may not affect protein function. Finally, 22 variants were classified as pathogenic or likely pathogenic. After evaluation of the clinical data and genetic evidence, the diagnoses of 31 patients from 25 families were confirmed. Genotype–phenotype correlation analysis from the cohort, consisting of 28 patients in this study and 235 published cases, showed that the onset of neurofibromas and bone lesions exhibited an age-dependent association, with 79.8% and 73.8% probability of developing in patients older than 23.5 years or 20.5 years, respectively. No association was found between the location or type of *NF1* variants and any specific features.

**Conclusions:**

We comprehensively described the clinical and genetic data of a Chinese NF1 cohort and emphasized the necessity of further functional analysis on splicing variants. Neurofibromas and bone lesions are age-dependent disease complications that exhibit progressive tendencies with increasing age in patients with NF1.

**Supplementary Information:**

The online version contains supplementary material available at 10.1186/s13023-025-03807-z.

## Background

Neurofibromatosis type 1 (NF1; MIM 162200) is an autosomal dominant disorder caused by germline variants of the *NF1* gene. It is one of the most prevalent genetic disorders, with an estimated incidence of 1 in 2500–3500 live births [1]. Genetic counseling for a patient with NF1 is challenging because of its highly variable phenotypic expressions, encompassing a spectrum from mild cutaneous manifestations to severe complications, such as central nervous system neoplasms [2]. Café-au-lait macules (CALMs), neurofibromas, freckling nodules, and Lisch nodules are prominent features of NF1. Additionally, individuals with NF1 may experience optic gliomas, specific bone lesions, and learning difficulties [3]. Notably, the incidence of cancer in NF1 patients is significantly higher than that in the general population [4]. To facilitate the diagnosis of NF1, the National Institute of Health (NIH) has established internationally accepted precise guidelines. However, children younger than eight years with no family history of the condition are unlikely to meet the minimum NIH criteria for NF1 because the prototypical features may not fully manifest until adulthood [5]. Consequently, molecular genetic testing has been incorporated into the diagnostic standards for NF1 confirmation, and it is invaluable in distinguishing NF1 from other conditions in young children [6].

With the increasing use of whole-exome sequencing (WES) in the clinical evaluation of patients with suspected NF1, an increasing number of sporadic pathogenic variants and associated phenotypes are being identified and reported. Given the high clinical variability and wide range of mutational heterogeneity in NF1, it is necessary to establish a genotype–phenotype correlation for this disorder [7]. Although previous studies have explored the relationship between specific variant types or locations and particular phenotypes [8–13], research on the incidence rate and onset age of individual NF1 features remains limited [14, 15]. This information is of great significance for prenatal genetic counseling and tailoring surveillance strategies for potentially treatable complications.

In this study, we conducted a comprehensive analysis of 27 Chinese pedigrees suspected of having NF1. Genetic diagnosis identified 10 previously unreported variants, expanding the known variant spectrum of *NF1*. Functional RNA analysis was performed on a canonical splicing variant to facilitate variant interpretation. Furthermore, we analyzed the genotype and phenotype correlation using patients in our cohort and those with clinical and genetic data accessible from the published literature. The prevalence of each clinical manifestation among patients with NF1 and its risk factors were assessed.

## Methods

### Patients in the Chinese cohort

Between January 2019 and June 2023, 27 families diagnosed or clinically suspected of having NF1 were referred to our hospital. Of these, 25 families presented with a confirmed diagnosis of NF1. Two additional families were referred, owing to a family history of tumors (Family 9) and adverse pregnancy outcomes (Family 10), of which both were identified with candidate *NF1* variants. The age of the patients in our cohort ranged from 2 to 78 years. A comprehensive review of patient medical records was performed to extract detailed information on their intellectual, musculoskeletal, neurological, ocular, and dermatological conditions.

### Whole exome sequencing, Sanger sequencing, and multiplex ligation-dependent probe amplification

Genomic DNA (gDNA) was extracted from blood samples using the QIAamp DNA Blood Mini Kit (Qiagen, Hilden, Germany). Targeted exome capture was performed using the KAPA HyperExome or HyperExplore MAX 3 Mb T1 (Roche, Basel, Switzerland). The captured libraries were sequenced on an MGISEQ-2000 using paired-end 100 reads (BGI, Shenzhen, China). The average sequencing depth of the target region was ≥ 180×, with over 96% of the loci achieving a coverage depth of > 20×. Sequencing data were mapped to the human reference genome (hg19) using SOAP and BWA, and variant calling was performed using the Genome Analysis Toolkit (GATK). All identified variants were annotated, filtered, and interpreted using the Sunburst Genetic Analysis and Interpretation Platform (BGI, Shenzhen, China) and Translational Genomic Expert (TGex). The transcript NM_000267.3 was employed for the annotation of *NF1* gene variants. Predictive tools, including REVEL (https://sites.google.com/site/revelgenomics/), MutationTaster (https://www.mutationtaster.org/), and CADD (http://cadd.gs.washington.edu), were used to assess the potential effects of the variants on the NF1 protein. The impact of variants on splicing was evaluated using in silico tools, including SpliceAI (https://spliceailookup.broadinstitute.org/) and varSEAK (https://varseak.bio/). Amino acid conservation in multiple species was analyzed using PhyloP software and the online website Clustal Omega (http://www.ebi.ac.uk/Tools/msa/clustalo/). The variants were classified according to the American College of Medical Genetics and Genomics (ACMG) guidelines [16].

Sanger sequencing was performed to validate candidate variants identified by WES and analyze segregation patterns in related NF1 cases. The primers used in this study are listed in Supplementary Table 1. In cases where WES failed to detect a causal variation, multiplex ligation-dependent probe amplification with P081 and P082 probe mixes (MRC-Holland, Amsterdam, Holland) was used following the manufacturer’s instructions.

### Functional analysis of the novel splicing variant

To assess the effect of the *NF1* splice site variant (*NF1* c.3497–2 A > G) on mRNA splicing, total blood RNA from suspected patients and their family members was isolated using the PaxGene Blood RNA Collection Kit (Qiagen, Hilden, Germany). Following the manufacturer’s instructions, 1 µg of RNA was reverse-transcribed. The region was amplified using Takara Taq DNA Polymerase in cDNA with the following primers: forward: 5′-TAGTTGAAGTAATGATGGCAAGGAG-3′ and reverse: 5′-CCAGGAGTTTTTGTAGATAGGTAGC-3′. The PCR products were separated on a 1% agarose gel and visualized using SYBR Safe DNA Gel Stain. The polymerase chain reaction (PCR) products were subjected to Sanger sequencing.

### Retrieval and extraction of patient data from the published literature

The correlation analysis encompassed patients with NF1 from our cohort and those extracted from a comprehensive review of the literature. Literature searches were conducted in PubMed and Embase databases using the following keywords: (“NF1” OR “Neurofibromatosis type 1”) AND (“variant” OR “mutation” OR “genotype”) AND (“Phenotype” OR “clinical manifestation” OR “clinical feature” OR “genotype-phenotype”). The search was restricted to articles published in English between 2013 and the date of the search (31 July 2024). Duplicates were manually removed using EndNote software (Version X9.0). Two authors (HJF and XH) independently reviewed the titles, abstracts, and keywords of the identified publications and selected relevant papers for in-depth examination. After a thorough evaluation, a study was included if it met the following criteria: [1] the study population consisted of patients with confirmed NF1 diagnoses, encompassing both genetic and clinical characteristics; and [2] for each patient, clinical phenotypic data included a minimum of five clinical manifestations from different organ systems associated with NF1, along with age, sex, and genetic details, as described in previous studies [17–20]. Studies that solely diagnosed NF1 based on phenotypic characteristics or those that exclusively presented patient causal variants without providing a detailed or specific clinical history were excluded. Conflicts were resolved through consensus or consultation with a senior investigator (SYL).

Of the initial 53 studies, four met the inclusion criteria and were selected for data extraction (Supplementary Fig. 1). The following details were extracted: genomic coordinates (GRCh37/hg19), exons or introns where the variant was located, nucleotide changes, amino acid changes, variant nomenclature for cDNA, and associated phenotypes.

### Statistical analysis

The frequency distribution of the clinical manifestations of the patients was analyzed using the chi-square goodness-of-fit test. The association between continuous and dichotomous variables was assessed using point-biserial correlation coefficients. Receiver operating characteristic (ROC) curves were constructed to evaluate the accuracy of the age of onset in predicting the occurrence of clinical phenotypes and determine the optimal cutoff value for onset age. Chi-square tests were used to analyze whether the position or type of variant was associated with specific phenotypes. The analyses were performed using SPSS Statistics v.25 and R software (v.4.3.1). A two-tailed *P* < 0.05 was considered statistically significant.

## Results

### Genetic results of the 27 families with suspected NF1 patients

**Fig. 1:**
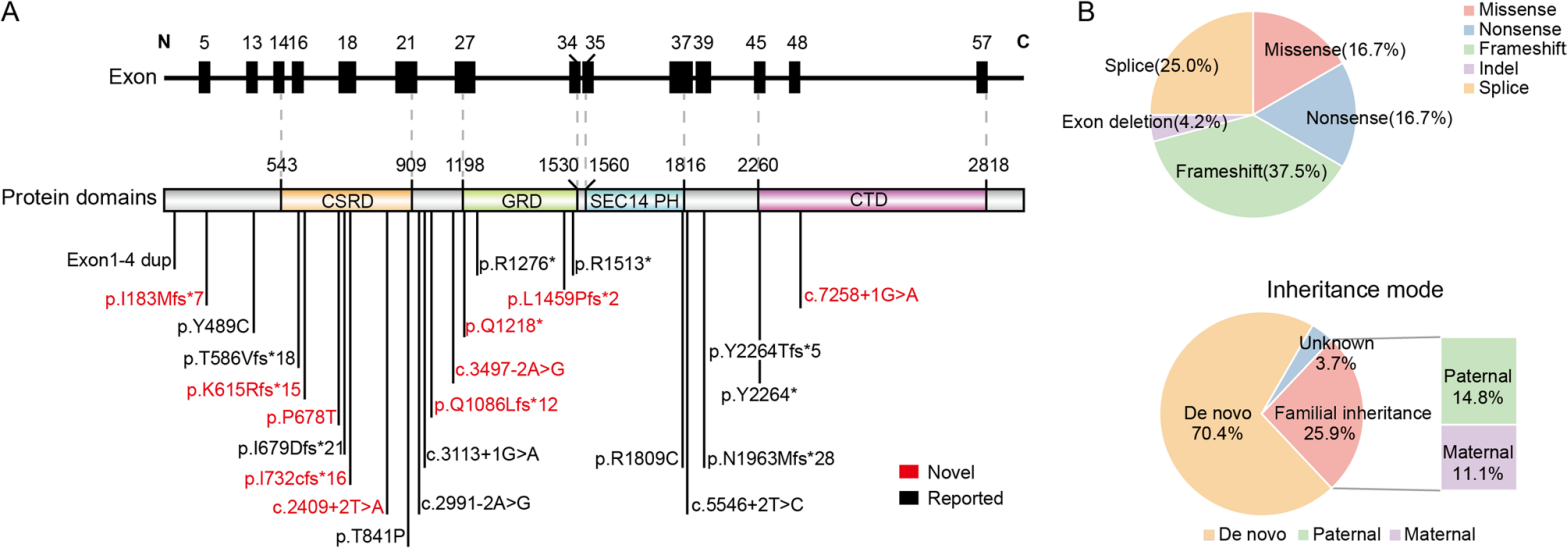
Spectrum, distribution, and basic features of *NF1* variants identified in our cohort. (**A**) The locations of 24 *NF1* gene variants are displayed, along with their distribution within the *NF1* domains. Variant positions are indicated by vertical lines, and novel *NF1* variants are highlighted in red. The relative positions of exons from the *NF1* gene are shown at the top with black bars (NM_000267.3). Known neurofibromin domains include CSRD (cysteine and serine-rich domain, residues 543–909), GRD (GTPase activating protein-related domain, residues 1198–1530), SEC14/PH (Sect14 and pleckstrin homologous domain, residues 1560–1816), and CTD (carboxy terminal domain, residues 2260–2818). (**B**) Upper panel: Distribution of *NF1* variants observed in our patients. Lower panel: The inheritance mode of *NF1* variants identified from 26 families. Inherited variants are shown in yellow, whereas *de novo* variants are displayed in blue

**Fig. 2:**
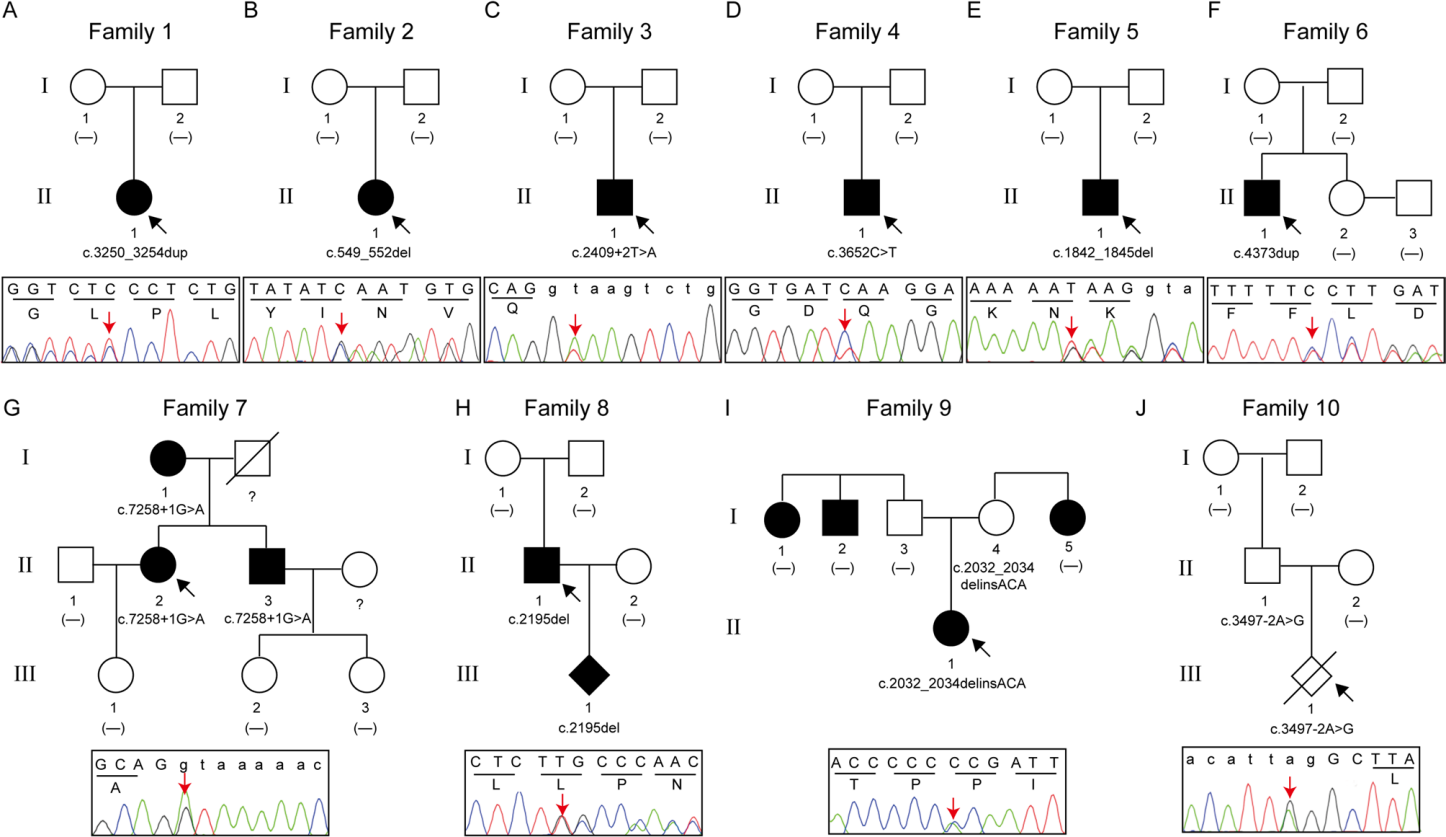
Pedigree charts and Sanger sequencing results of 10 families with novel *NF1* variants identified in this study. Squares and circles denote males and females, respectively; unfilled and filled symbols denote unaffected individuals and affected individuals, respectively. The arrows point to the probands

Genetic analysis of 101 individuals (including 33 suspected patients) from 27 families revealed 24 distinct heterozygous *NF1* variants, including 9 (37.5%) frameshift, 6 (25.0%) splice site, 4 (16.7%) nonsense, 4 (16.7%) missense, and 1 (4.2%) exon deletion mutations (Table 1; Fig. 1A and B). Among the 27 families, 19 (70.4%) had *de novo NF1* variants, 7 (25.9%) were familial inheritance, and 1 (3.7%) could not be determined owing to the unavailability of parental samples (Fig. 1B, lower panel). Of the identified variants, 10 were novel that have not been previously reported, and the pedigrees of the 10 families are shown in Fig. 2.

**Table 1:** Characteristics and classifications of *NF1* variants identified in this study

Family	Mutations	Exons	AA Substitutions	Allele frequency in gnomAD	Clinical significance in Clinvar	LOVD	De novo	Evidence criterion	ACMG classification	Reported in literature
1	c.3250_3254dup	25	p.(Gln1086Leufs*12)	—	—	—	Yes	PVS1, PM2_Supporting, PM6	Pathogenic	No
2	c.549_552del	5	p.(Ile183Metfs*7)	—	—	—	Yes	PVS1, PM2_Supporting, PM6	Pathogenic	No
3	c.2409 + 2T > A	/	/	—	Pathogenic (One star)	Pathogenic (NF1_000206)	Yes	PVS1_Strong, PM2_Supporting, PM6	Likely pathogenic	No
4	c.3652 C > T	27	p.(Gln1218*)	—	Pathogenic (One star)	—	Yes	PVS1, PM2_Supporting, PM6	Pathogenic	No
5	c.1842_1845del	16	p.(Lys615Argfs*15)	—	—	—	Yes	PVS1, PM2_Supporting, PM6	Pathogenic	No
6	c.4373dup	33	p.(Leu1459Profs*2)	—	—	—	Yes	PVS1, PM2_Supporting, PM6	Pathogenic	No
7	c.7258 + 1G > A	/	/	—	—	Pathogenic (NF1_001587)	No	PVS1_Strong, PM2_Supporting, PP1	Likely pathogenic	No
8	c.2195del	18	p.(Leu732Cysfs*16)	—	—	—	Yes	PVS1, PM2_Supporting, PM6	Pathogenic	No
9	c.2032_2034delCCGinsACA	18	p.(Pro678Thr)	0.00002793	Uncertain significance (One star)	—	No	PM2_Supporting, PP2, BP3	Uncertain significance	No
10	c.3497–2 A > G^#^	/	/	—	Uncertain significance (One star)	—	Yes	PVS1_Moderate, PM2_Supporting	Uncertain significance	No
11	c.2521 A > C	21	p.(Thr841Pro)	—	Pathogenic/Likely pathogenic (Two star)	—	Yes	PS2_VeryStrong, PM2_Supporting, PP2, PP3	Pathogenic	Yes, < 5 times
12	c.6787_6790delACTT	45	p.(Tyr2264Thrfs*5)	—	Pathogenic (Two star)	—	Yes	PVS1, PM2_Supporting, PS2, PS4_Moderate	Pathogenic	Yes, > 5 times
13	c.1466 A > G	13	p.(Tyr489Cys)	0.00001197	Pathogenic (Two star)	Pathogenic (NF1_000063)	No	PS3_Supporting, PS4_Strong, PM2_Supporting, PM6, PP2, PP4	Pathogenic	Yes, > 5 times
14	c.4537 C > T	34	p.(Arg1513*)	—	Pathogenic/Likely pathogenic (Two star)	Pathogenic (NF1_000816)	Yes	PVS1, PM6_Strong, PS4, PP4	Pathogenic	Yes, > 5 times
15	c.5546 + 2T > C	/	/	—	—	Pathogenic (NF1_001573)	Yes	PVS1, PM2_Supporting, PM6, PS4_Supporting	Pathogenic	Yes, < 5 times
16, 26	c.6792 C > A	45	p.(Tyr2264*)	—	Pathogenic (Two star)	Pathogenic (NF1_000816)	/, No	PVS1, PM2_Supporting, PM6, PS4	Pathogenic	Yes, > 5 times
17, 18, 21	c.1754_1757delTAAC	16	p.(Thr586Valfs*18)	—	Pathogenic (Two star)	—	Yes, Yes, Yes	PVS1, PM2_Supporting, PS2, PS4	Pathogenic	Yes, > 5 times
19	c.2991–2 A > G	/	/	—	Pathogenic/Likely pathogenic (Two star)	Pathogenic (NF1_000289)	Yes	PVS1_Strong, PM2_Supporting, PS4, PM6, PP4	Pathogenic	Yes, > 5 times
20	c.2033dup	18	p.(Ile679Aspfs*21)	—	Pathogenic (Two star)	Pathogenic (NF1_000148)	No	PVS1, PM2_Supporting, PS4	Pathogenic	Yes, > 5 times
22	c.(?_-383-1)_(479 + 1_480-1)dup /Exon1-4dup	1–4	p.(?)	—	—	—	No	PVS1_Strong, PM2_Supporting, PP4	Likely pathogenic	Yes, < 5 times
23	c.3113 + 1G > A	/	/	0.000003987	Pathogenic (Two star)	Pathogenic (NF1_000306)	Yes	PVS1, PS3_Supporting, PS4_Strong, PM2_Supporting, PM6	Pathogenic	Yes, > 5 times
24	c.3826 C > T	28	p.(Arg1276*)	0.000003981	Pathogenic (Two star)	Pathogenic (NF1_000403)	Yes	PVS1, PS4_Moderate, PM2_Supporting	Pathogenic	Yes, > 5 times
25	c.5425 C > T	37	p.(Arg1809Cys)	—	Pathogenic (Two star)	Pathogenic (NF1_000653)	No	PS4, PS3_Supporting, PM2_Supporting, PP1_Strong, PM6, PP2, PP3	Pathogenic	Yes, > 5 times
27	c.5888del	39	p.(Asn1963Metfs*28)	—	—	—	Yes	PVS1, PM2_Supporting, PS2	Pathogenic	Yes, < 5 times

^#^ Functional assays revealed that the c.3497–2 A > G variant results in a 6-bp in frame deletion (c.3497_3502del, p.Leu1167_Gly1168del)

Upon re-evaluation of the 24 variants and integration of clinical data, 22 were classified as pathogenic or likely pathogenic, and two (c.2032_2034delCCGinsACA and c.3497–2 A > G) were categorized as variant of uncertain significance (VUS) (Table 1). The c.2032_2034delCCGinsACA variant, identified in a sporadic case from Family 9 with a family history of cancer, was a missense variant (p.Pro678Thr). Despite being a rare variant, it was predicted to be benign, and family segregation analysis indicated a lack of co-segregation with the disease. Therefore, it was interpreted as VUS-favor benign. The c.3497–2 A > G variant, identified incidentally through WES in a case of induced fetal labor with cleft lip and palate in Family 10, was also detected in the fetus’s father who did not show any symptoms of NF1 (Fig. 2J). In silico analysis showed that the c.3497–2 A > G variant might affect the canonical splice site and was predicted to be “deleterious” (Supplementary Fig. 2A). However, the functional analysis showed that the c.3497–2 A > G variant activated a new cryptic 3′ acceptor splice site within exon 27, resulting in a 6-bp in-frame deletion of the *NF1* gene. The mRNA level of *NF1* in the carrier was comparable to the healthy control (Supplementary Fig. 2B-D). Consequently, this variant was classified as VUS.

### Clinical characteristics of patients

In addition to unclear molecular diagnostic conclusions, the clinical characteristics of the patients in families 9 and 10 also do not meet the NF1 diagnostic criteria. Family 9 only had a family history of cancer, and the *NF1* variant was an incidental finding in Family 10. Finally, 31 patients from 25 families were definitely diagnosed with NF1. Among them, one fetus was induced labor (Family 27) and two families were lost to follow up (Family13 and Family 19). We therefore collected and analyzed the clinical data of 28 patients from 22 families. As shown in Table 2, CALMs were observed in 27 (96.4%) patients, and 18 (64.3%) patients had a cutaneous neurofibromas phenotype, with eight occurring sporadically and 10 appearing in multiple instances. The prevalence of optic gliomas is unknown because most patients do not undergo formal ophthalmological examinations, but we noticed that half of the patients had myopia. Bone lesions (mainly scoliosis) were observed in 11 (39.3%) patients, and neurological manifestations, such as learning problems and intellectual disability were identified in 6 (21.4%) patients. Punctate brain tumors or patchy tumor signals occurred in 5 (17.9%) patients and 1 (3.6%) patient experienced early-onset tumor.

**Table 2:** Clinical profile of the 28 NF1 patients

Family ID	Gender	Age (year)	Height (cm)	CALMs	Neurofibromas*	Myopia	Bone lesions	ID	Brain MR findings	Malignancy
1	Female	12	145	+	2 in the back of the neck)	-	-	-	Brain patchy signals	-
2	Female	11	152	+	3–4 in the back of the neck	+	40-degree scoliosis (with 1 neurofibroma)	+	Brain punctate tumor	-
3	Male	22	173	+	1 in thigh, 2 in back, 1 in heel, 1 in mediastinum	+	-	-	-	-
4	Male	15	172	+	Beaded around the neck	-	-	-	-	-
5	Male	14	160	+	-	+	55-degree scoliosis (with 1 neurofibroma)	+	Brain punctate tumor	-
6	Male	16	162	+	1 in arm	-	Mild scoliosis (with 1 neurofibroma)	-	-	-
7	Female	78	155	+	Distributed all over the body	-	-	-	-	-
	Female	56	158	+	Distributed all over the body	-	Mild scoliosis	-	-	-
	Male	58	171	+	Distributed all over the body	-	-	-	-	-
8	Male	33	177	+	> 10 distributed over the body	+	-	-	-	-
11	Male	7	118	+	-	+	-	+	-	-
12	Female	36	160	+	Distributed all over the body	+	50-degree scoliosis (with 1 neurofibroma)	-	-	-
14	Female	17	157	+	-	+	-	-	-	-
15	Female	11	145	+	1 on the back	+	Mild scoliosis	+	Brain patchy signals	-
16	Female	37	160	+	Distributed all over the body	-	-	-	-	-
17	Female	8	130	+	1 on the feet	+	-	+	-	-
18	Male	17	176	+	-	+	leg was not straight	+	-	-
20	Female	37	150	+	on the left leg	+	Mild scoliosis	-	-	-
	Male	66	171	+	Distributed all over the body	-	Mild scoliosis	-	-	-
	Female	63	151	+	Distributed all over the body	-	> 30-degree scoliosis	-	-	-
21	Male	34	175	+	1 on the waist	+	> 50-degree scoliosis (with 1 neurofibroma)	-	-	-
22	Male	8	126	+	-	-	-	-	-	-
	Male	37	178	-	-	-	-	-	-	-
23	Male	10	145	+	-	-	-	-	Brain patchy signals	-
24	Male	7	110	+	-	-	-	-	-	leukemia
25	Male	34	161	+	-	+	-	-	-	-
	Male	61	150	+	-	-	-	-	-	-
26	Female	37	165	+	Distributed all over the body	+	-	-	-	-
Sum				27/28 (96.4%)	18/28 (64.3%)	14/28 (50.0%)	11/28 (39.3%)	6/28 (21.4%)	5/28 (17.9%)	1/28 (3.6%)

* Neurofibromas represents a combination of both plexiform and cutaneous neurofibromas

Abbreviation: CALMs, café-au-lait macules; ID, intellectual disability

### Prevalence of each clinical manifestation and risk factors for NF1

**Fig. 3:**
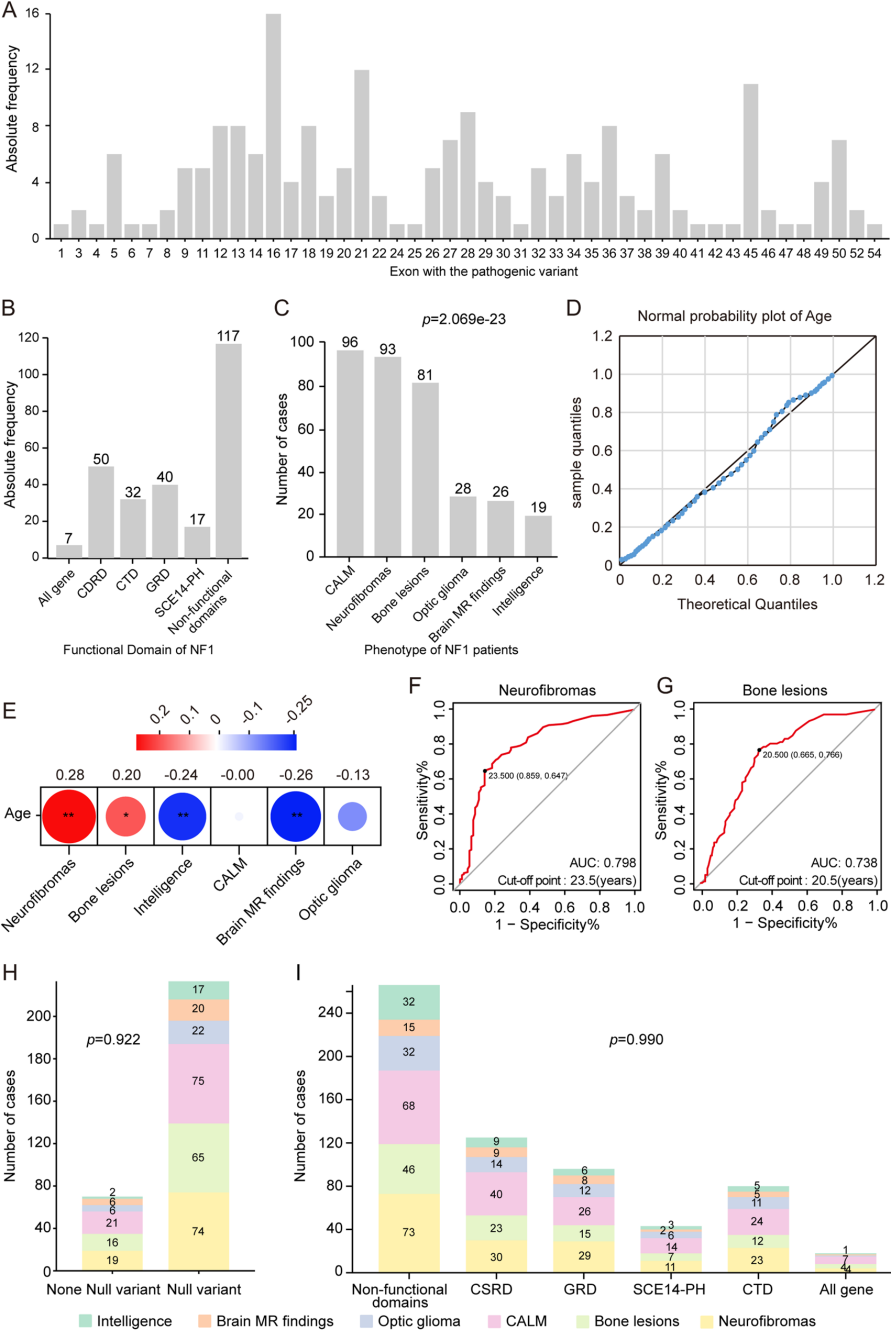
Genotype–phenotype correlation analysis in 263 patients with neurofibromatosis type 1 from our cohort and four literature databases. (**A**) Distribution of variants across the *NF1* exon. (**B**) Frequency of variations within functional domains. (**C**) Prevalence of various phenotypes among patients with neurofibromatosis type 1. (**D**) Normal probability plot according to age distribution. (**E**) Point-biserial correlation coefficient analysis revealed significant positive associations between age and the onset of neurofibromas phenotype and bone lesions. (**F**) Optimal threshold values for predicting neurofibromas onset were determined using ROC curve analysis. (**G**) Optimal threshold values for predicting bone lesion onset determined using ROC curve analysis. (**H**) Distribution of six phenotypes among different types of variants. (**I**) Distribution of six phenotypes among different domains of the NF1 protein. ROC, receiver operating characteristic; NF1, neurofibromatosis type 1. Neurofibromas represents a combination of both plexiform and cutaneous neurofibromas

To further analyze the phenotypic features of NF1 patients, we expanded our cohort by incorporating 235 NF1 patients from four published studies [17–20], all of which provided comprehensive phenotypic data (Supplementary Table 2). This cohort comprised 263 patients with 188 unique *NF1* variants, including 153 null variants and 36 variants of other types. We analyzed the pathogenicity of all putative missense and splicing variants using multiple in silico prediction tools, and found that all variants met the expected criteria for functional deleteriousness (Supplementary Table 3). Notably, 38 of the 188 variants were recurrent but scattered and random (Supplementary Table 4), whereas others were private (*n* = 150). Importantly, 188 variants were almost uniformly distributed throughout all exons, without hotspots (Fig. 3A). Similarly, these variants were not enriched in the specific functional domains of *NF1* (Fig. 3B).

To analyze the prevalence of the six phenotypic manifestations in patients with NF1, a chi-square goodness-of-fit test was conducted on 112 patients with complete data for the six phenotypes. The most prevalent phenotypes were CALMs (85.7%, 96/112), followed by neurofibromas (83.0%, 93/112), bone lesions (72.3%, 81/112), optic gliomas (25.0%, 28/112), brain magnetic resonance imaging findings (23.2%, 26/112), and intellectual disability (17.0%, 19/112). There was a significant difference in the incidence of the six phenotypes in these patients (*p* = 2.069e-23; Fig. 3C). Two-tailed chi-square test was used to assess co-occurrence relationship between the three most prevalent phenotypes and significant co-occurrence was observed only between neurofibromas and bone lesions (Supplementary Table 5).

Further analyses were conducted to explore the associations between phenotypes with age of onset, variant types, and variant locations. A normal probability plot revealed that the age at onset followed a normal distribution (Fig. 3D). The point biserial correlation analysis for age and phenotype in the 263 patients indicated a significant positive correlation between neurofibromas (*r* = 0.28, *p* = 3.234e-92) and bone lesions (*r* = 0.20, *p* = 3.320e-47) with age, suggesting a progressive trend for these two phenotypes with increasing age (Fig. 3E). This association led us to investigate whether age could serve as a significant predictor of neurofibroma and bone lesion onset in patients with NF1. The ROC curves for age, predicting the onset of neurofibromas and bone lesions, yielded area under the curve values of 0.798 (Fig. 3F) and 0.738 (Fig. 3G), respectively. Specifically, patients older than 23.5 years had a 79.8% likelihood of developing neurofibromas (Fig. 3F), whereas patients older than 20.5 years had a 73.8% likelihood of acquiring bone lesions (Fig. 3G). No significant differences were observed in the phenotypic distribution between null and other variants (Fig. 3H). Furthermore, there was no discernible difference in the phenotypic distribution based on the location of the variants within the functional domains (Fig. 3I).

## Discussion

Loss of function is a primary genetic mechanism for NF1 inactivation [21]. Consequently, assessing the pathogenicity of most null variants, such as frameshift and non-nonsense variants, is relatively straightforward. For splicing variants, especially canonical ± 1 or 2 splice sites, exon skipping is the most common consequence. However, caution should be exercised when relying solely on predicted outcomes. For example, the *NF1* variant c.3497–2 A > G, located at the classical splicing site, may induce the skipping of exon 27, leading to frameshift alterations and activation of nonsense-mediated mRNA decay. However, functional studies revealed that the c.3497–2 A > G variant activated a new cryptic 3′ acceptor splice site within exon 27, resulting in an in-frame alternation with a 6 bp deletion. Hence, the c.3497–2 A > G variant was more appropriately classified as a VUS (PVS1_Moderate, PM2_Supporting). Our results underscore the importance of functional studies to elucidate the functional consequences of splice variants. Recent recommendations for interpreting splicing variants also demonstrate this notion, emphasizing that the strength of PVS1 depends on the knowledge of functional relevance, the extent of protein loss, or both, with functional studies deemed necessary, especially when predictions are less confident [22].

The interpretation of missense variants poses an even greater challenge than the interpretation of splicing variants [23]. Timely dissemination of information on novel *NF1* variants is essential for pathogenicity classification [24]. For instance, the *NF1* variant c.1466 A > G was initially classified as VUS but was later reclassified as “pathogenic” owing to its recurrent occurrence in patients with NF1 [25, 26]. Furthermore, this study highlights the inherent limitations of in silico prediction tools, as evidenced by inconsistencies among results from different platforms. This variability is expected, considering the substantial size of a protein such as NF1, which complicates accurate computational modeling. Consequently, in silico predictions are typically employed as supplementary evidence in pathogenicity assessments. Following ACMG guidelines [27], we implemented the PP3 criterion for missense variants demonstrating either a REVEL score > 0.75 or concordant deleterious predictions from ≥ 2 of 3 computational tools. Additionally, segregation data from pedigree analysis also contributed to the classification of pathogenicity. Our findings confirm the limitations of in silico pathogenicity prediction and highlight the importance of reported cases and pedigree segregation analysis in the interpretation of *NF1* variants.

Several studies have investigated genotype–phenotype correlations among patients with *NF1* variants. *NF1* microdeletions are often associated with a severe clinical phenotype [8], whereas the variant c.2970_2972del (p.Met992del) is associated with a mild phenotype lacking externally visible plexiform, cutaneous, or subcutaneous neurofibromas [9]. Individuals with missense variants that affect the *NF1* codons 844–848 have a statistically higher risk of developing spinal neurofibromas, plexiform neurofibromas, and optic pathway gliomas, whereas the pathogenic recurrent missense variants of p.Met1149, p.Arg1276, and p.Lys1423 are correlated with the Noonan-like phenotype [12]. The p.Arg1830Cys variant usually results in a mild phenotype with major features consisting of pigmentary signs without neurofibromas or other NF1-related malignancies [10]. Additionally, the p.Arg1038Gly variant has been associated with a cutaneous phenotype without neurofibromas or other complications [13]. Although these results provide important information for genetic counseling of patients with specific variant types or locations, research on the disease progression and incidence rate of specific phenotypes in the entire population of patients with NF1 is still limited.

To expand upon these findings, we incorporated patients from the published literature into our cohort, focusing on those with complete clinical phenotype data (including at least five clinical manifestations involving different organ systems associated with NF1). We found that the three most prevalent phenotypes in patients with NF1 were CALMs (85.7%), neurofibromas (83.0%), and bone lesions (72.3%). A significant difference was observed in the incidence of different phenotypes (*p* = 2.069e-23), which was consistent with previous reports [28]. We here observed a notably lower incidence of neurofibromas compared to the rates reported in prior studies, which have suggested that nearly 99% of adults with NF1 develop cutaneous neurofibromas [29]. We speculate this was related to age since the incidence rate of neurofibromas in adult patients was 80% (12/15) while in pediatric patients was only 46.2% (6/13) in our own cohort. Based on these considerations, we systematically re-analyzed and redefined the phenotypic spectrum in adult NF1 patients. The incidence of most prevalent phenotypes in 99 adult patients was neurofibromas (88.9%), CALMs (83.8%) and bone lesions (77.8%) (Supplementary Fig. 3A). The lack of statistically significant genotype–phenotype correlations in the adult cohort (Supplementary Fig. 3B-C) supports the hypothesis that this relationship remains age-independent within the NF1 population. On the other hand, incomplete collection of clinical data reported in the literature might also contribute to the observation of lower incidence of neurofibromas, as evidenced by the incidence of CALM. In our own cohort, the incidence of CALM was 96.4% (27/28), closely aligning with the 99% reported in published cohorts (Data from GeneReviewers, https://www.ncbi.nlm.nih.gov/books/NBK1109/). However, this rate decreased to 85.7% (96/112) after incorporating the literature cohort.

Noteworthy, Our study revealed that the clinical characteristics were age-dependent, particularly for neurofibromas and bone lesions. A progressive tendency was observed in these phenotypes with increasing age, suggesting that age can serve as a significant predictor of neurofibromas and bone lesion onset in patients with NF1. Patients older than 23.5 years had a 79.8% probability of developing neurofibromas, whereas those older than 20.5 years had a 73.8% likelihood of acquiring bone lesions. This critical information should be considered when developing follow-up strategies. These results provide new insights into the overall progression of NF1 as well as more effective digital information for genetic counseling, which will contribute to the long-term management of patients in clinical practice. For example, more attention is needed to neurofibromas and bone lesion phenotypes for NF1 patients beyond 23.5 years or 20.5 years. No apparent association between phenotypic distribution and variant types or locations was identified.

## Conclusions

In conclusion, this study identified 10 novel *NF1* variants, expanding the spectrum of *NF1* variations. The pathogenicity analysis of these variants underscores the significance of functional experiments, pedigree analysis, and data sharing for a comprehensive evaluation of *NF1* variations pathogenicity. Through a comprehensive analysis of potential genotype–phenotype correlations in an expanded cohort of 263 NF1 patients, we found that neurofibromas, bone lesions, and CALMs were the most prevalent phenotypes. A substantial association between the onset of neurofibromas and bone lesions and increasing age was identified. These results offer valuable information for healthcare professionals, enabling them to tailor patient follow-up strategies based on an individualized risk assessment.

## Electronic supplementary material

Below is the link to the electronic supplementary material.

Supplementary Material 1

Supplementary Material 2

Supplementary Material 3

Supplementary Material 4

## Data Availability

The raw data supporting the conclusions of this manuscript will be made available by the authors, without undue reservation, to any qualified researcher.

## Abbreviations

NF1: Neurofibromatosis type 1
WES: Whole exome sequencing
NIH: National Institutes of Health
CALMs: Café-au-lait macules
GTP: Guanosine triphosphate
