# Supplementary figures and images for: Genetic analysis of 25 Chinese pedigrees with neurofibromatosis type 1 and genotype-phenotype study from an extended cohort
Source: Orphanet J Rare Dis. 2025 May 23;20:246. doi: 10.1186/s13023-025-03807-z (PMC12102882; doi:10.1186/s13023-025-03807-z)

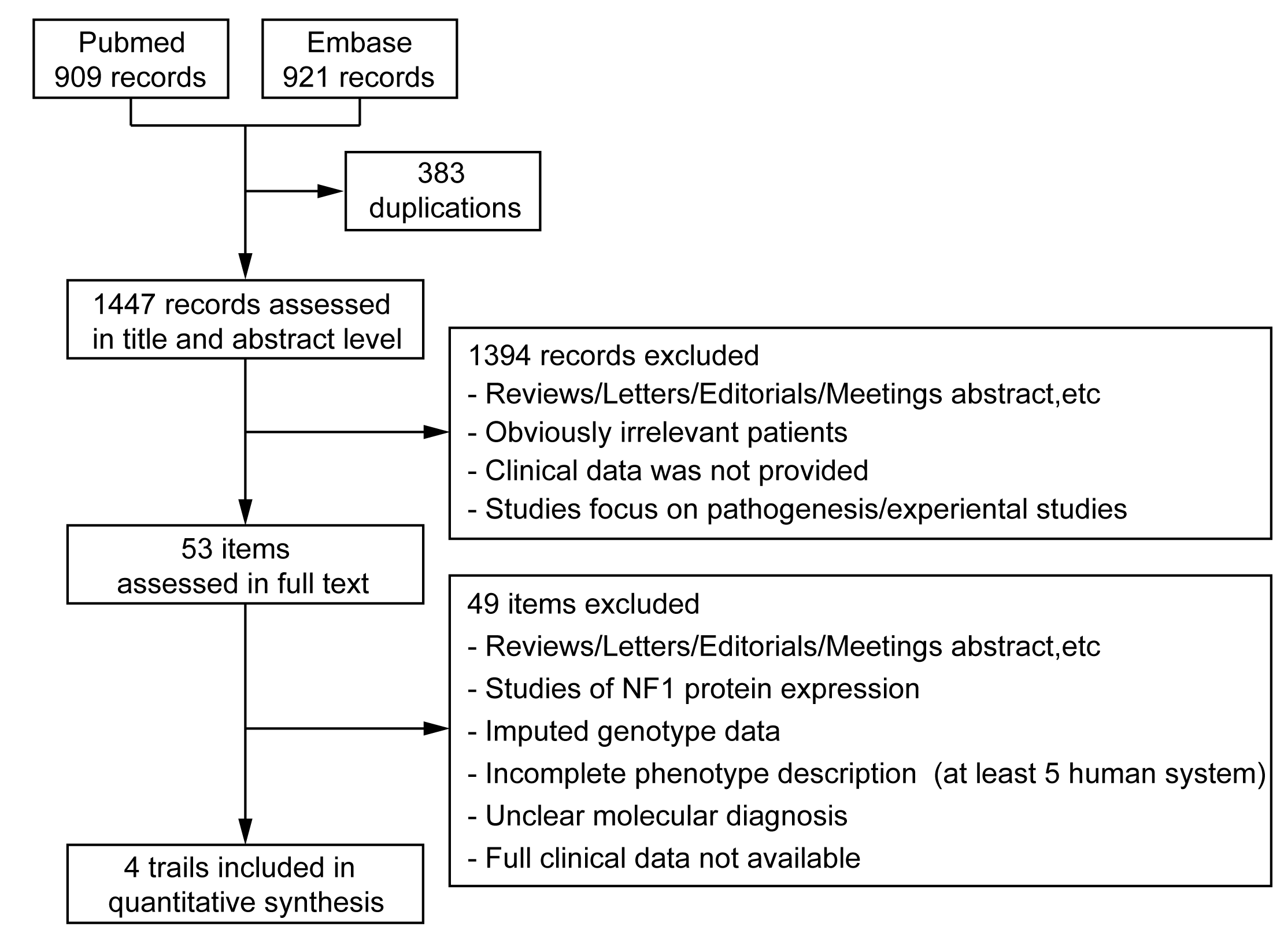

Supplement: Supplementary file 2 — Supplementary Material 2 [file 13023_2025_3807_MOESM2_ESM.tif]

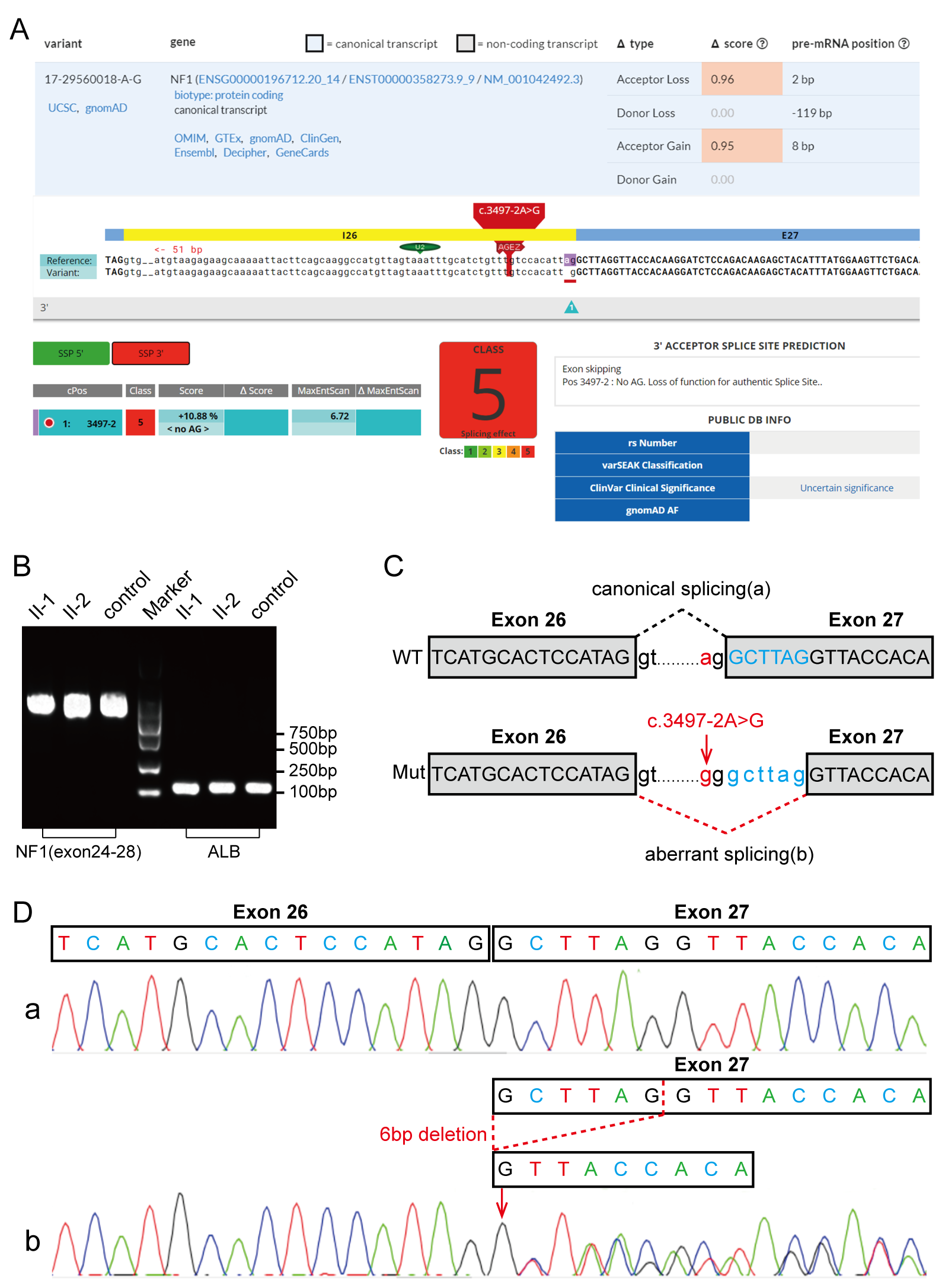

Supplement: Supplementary file 3 — Supplementary Material 3 [file 13023_2025_3807_MOESM3_ESM.tif]

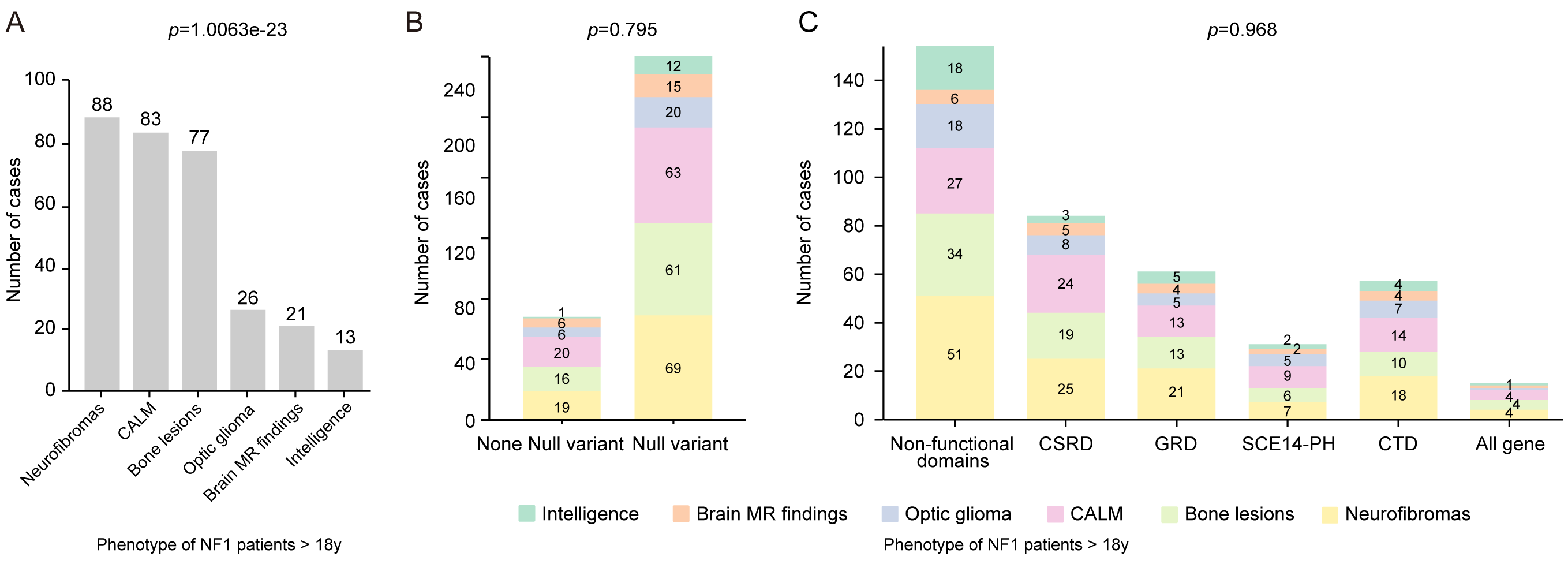

Supplement: Supplementary file 4 — Supplementary Material 4 [file 13023_2025_3807_MOESM4_ESM.tif]
